# Supplementary material for: Enhanced warming of European mountain permafrost in the early 21st century
Source: Nat Commun. 2024 Dec 10;15:10508. doi: 10.1038/s41467-024-54831-9 (PMC11631975; doi:10.1038/s41467-024-54831-9)
Supplement: Supplementary file 1 — Supplementary Information [file 41467_2024_54831_MOESM1_ESM.pdf]

Supplementary Information

Enhanced warming of European mountain permafrost in the early 21st century

Jeannette Noetzli[1,2], Ketil Isaksen[3], Jamie Barnett[4], Hanne H. Christiansen[5], Reynald Delaloye[6], Bernd Etzelmüller[7], Daniel Farinotti[8,9], Thomas Gallemann[10], Mauro Guglielmin[11], Christian Hauck[6], Christin Hilbich[6], Martin Hoelzle[6], Christophe Lambiel[12], Florence Magnin[13], Marc Oliva[14], Luca Paro[15], Paolo Pogliotti[16], Claudia Riedl[17], Philippe Schoeneich[18], Mauro Valt[19], Andreas Vieli[20], Marcia Phillips[1,2]*

1 *WSL Institute for Snow and Avalanche Research SLF, Davos Dorf, Switzerland*
2 *Climate Change, Extremes and Natural Hazards in Alpine Regions Research Centre CERC, Davos Dorf, Switzerland*
3 *Norwegian Meteorological Institute, Oslo, Norway*
4 *Department of Geological Sciences, Stockholm University, Stockholm, Sweden*
5 *Arctic Geophysics Department, University Centre in Svalbard, Longyearbyen, Norway*
6 *Department of Geosciences, University of Fribourg, Fribourg, Switzerland*
7 *Department of Geosciences, University of Oslo, Oslo, Norway*
8 *Laboratory of Hydraulics, Hydrology and Glaciology (VAW), ETH Zurich, Switzerland*
9 *Swiss Federal Institute for Forest, Snow and Landscape Research WSL, Birmensdorf, Switzerland*
10 *Bavarian Environment Agency, Augsburg, Germany*
11 *Department of Theoretical and Applied Science, Insubria University, Italy*
12 *Institute of Earth Surface Dynamics, University of Lausanne, Lausanne, Switzerland*
13 *Laboratoire EDYTEM, CNRS/Université Savoie Mont-Blanc, Le Bourget-du-Lac, France*
14 *Department of Geography, Universitat de Barcelona, Barcelona, Spain*
15 *Environmental Protection Agency of Piedmont, Turin, Italy*
16 *Environmental Protection Agency of Valle d'Aosta, Saint Christophe, Italy*
17 *GeoSphere Austria, Salzburg, Austria*
18 *PACTE, Institut d'Urbanisme et de Géographie Alpine, Université Grenoble Alpes, Grenobles, France*
19 *Environmental Protection Agency of Veneto, Centro Valanghe di Arabba, Arabba, Italy*
20 *Department of Geography, University of Zurich, Zurich, Switzerland*

** Corresponding author: Jeannette Noetzli, email: jeannette.noetzli@slf.ch, phone: +41 81 417 0375*

Content: Supplementary Figures 1–9

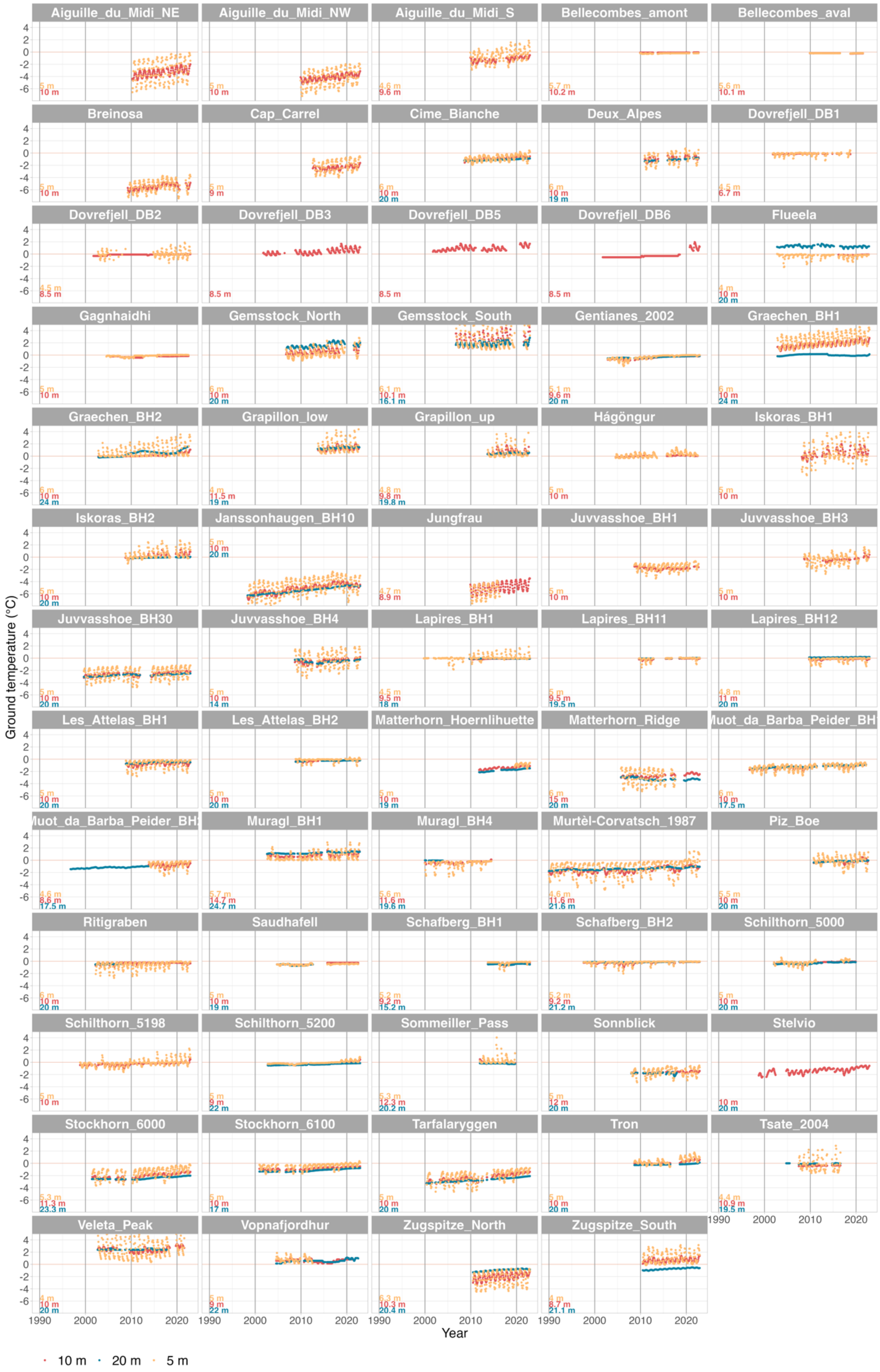

Supplementary Fig. 1. Monthly ground temperatures for 64 boreholes in European mountain permafrost regions measured at 5, 10, and 20 m depth. The exact depths of the sensors are given in the lower left corner of each facet. Monthly ground temperatures were aggregated based on daily values.

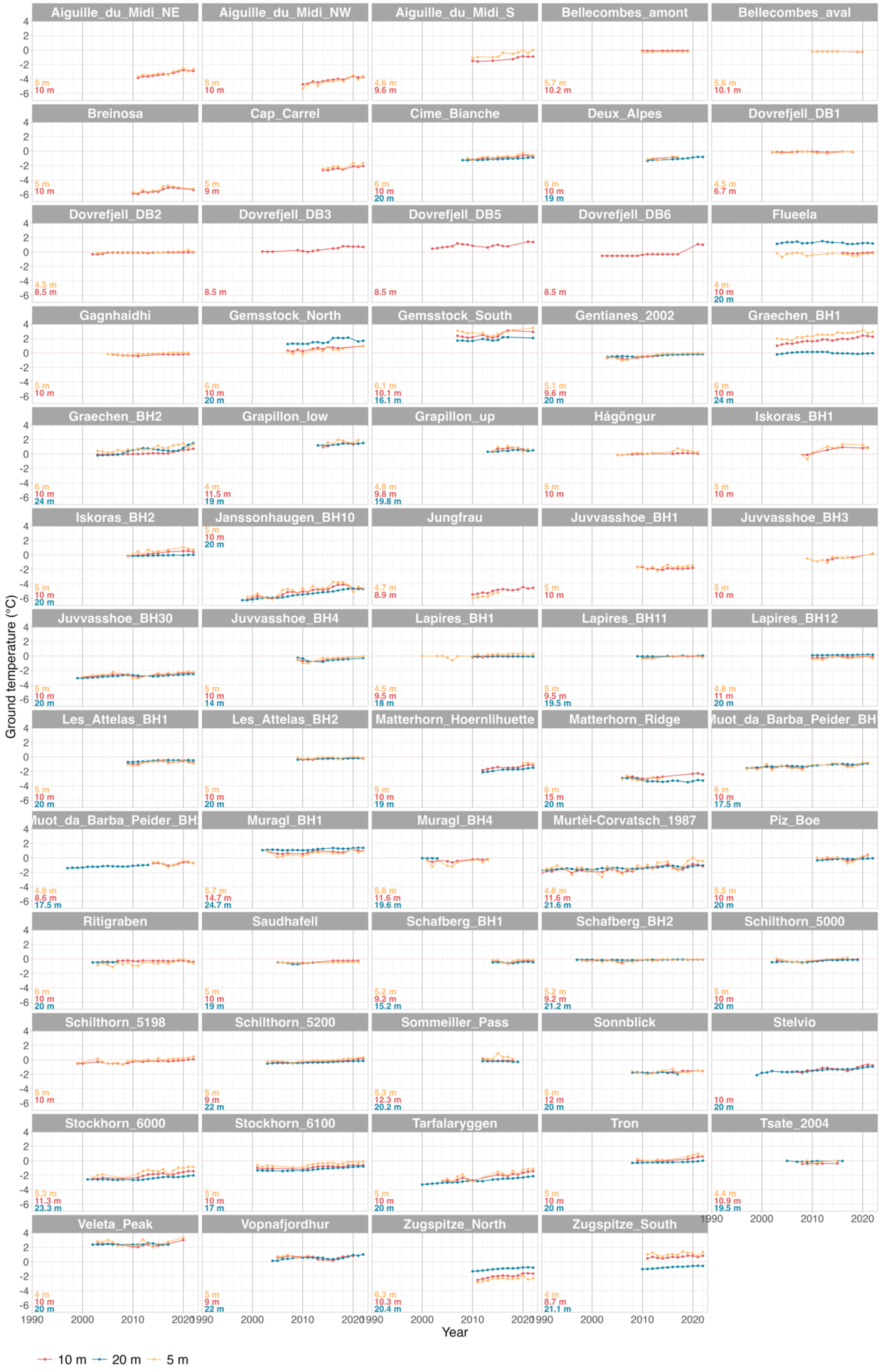

Supplementary Fig. 2. Annual ground temperatures for 64 boreholes in European mountain permafrost regions measured at 5, 10, and 20 m depth. The exact depths of the sensors are given in the lower left corner of each facet. Annual ground temperatures were aggregated based on monthly values (cf. Supplementary Fig. 1).

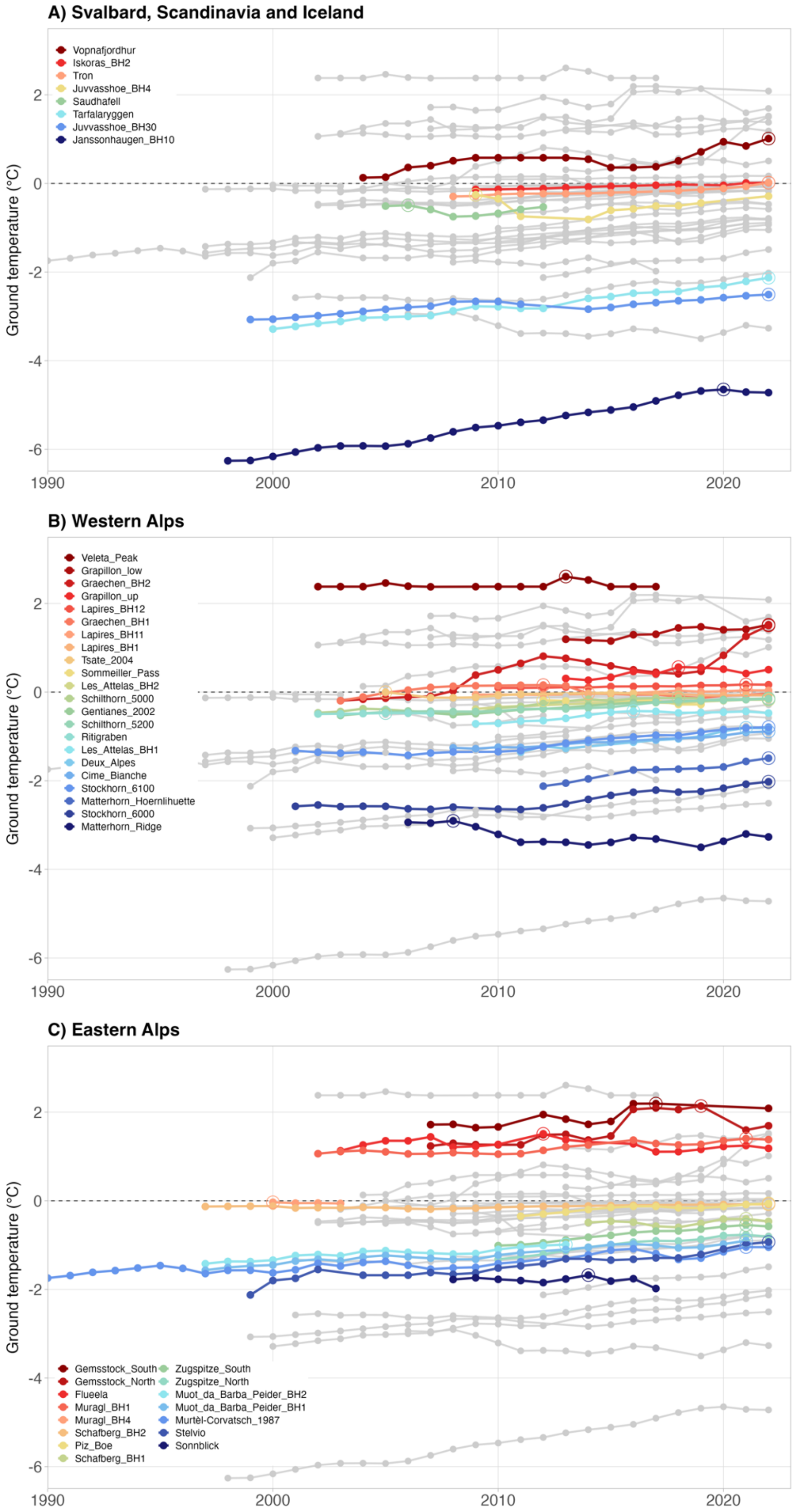

Supplementary Fig. 3. Fourty-five records of annual ground temperatures obtained in or next to permafrost areas in European mountains at approximately 20 m depth that cover at least one decade. a Svalbard, Scandinavia (sites in mainland Norway and Sweden) and Iceland, **b** Western Alps (sites in France, Western Switzerland and North-western Italy, and including the site in the Spanish Sierra Nevada), and **c** Eastern Alps (sites in Central and Eastern Switzerland, North-eastern Italy, Germany and Austria). The circles indicate the maximum value of each time series. In each panel, the time series of the other regions are shown in grey for comparison.

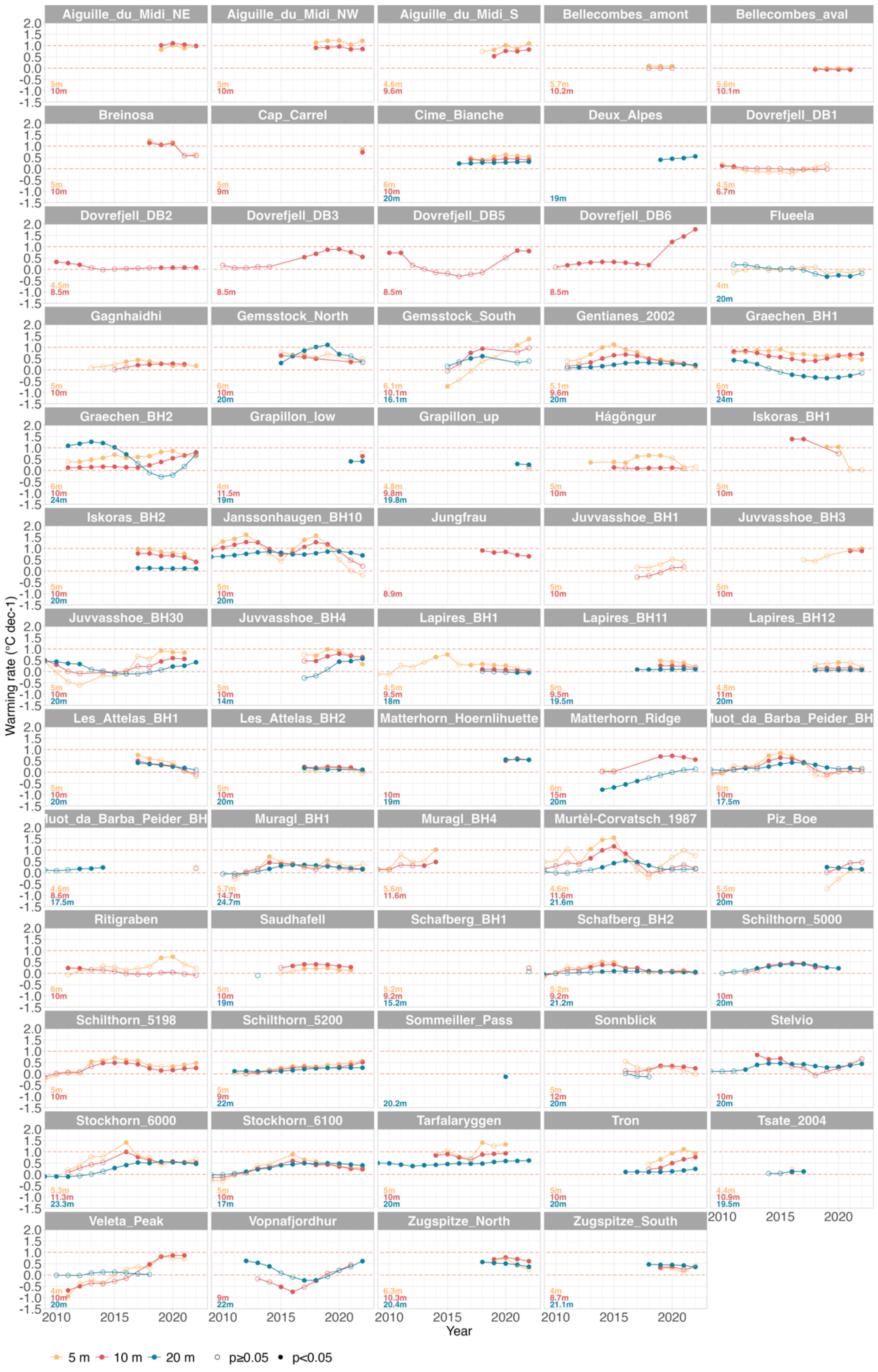

Supplemenary Fig. 4. Running warming rates for 64 ground temperature time series in or close to permafrost in European mountains calculated with an averaging window of 10 years. Depths of 5, 10 and 20 m are distinguished by colours, significance of the warming rates at 0.05 level is distigiuished by open or solid circle symbols. Values are plotted at the end of the period, e.g. warming rates for 2013–2022 are shown at x=2022. Exact depths of the sensors are given at the bottom of each plot.

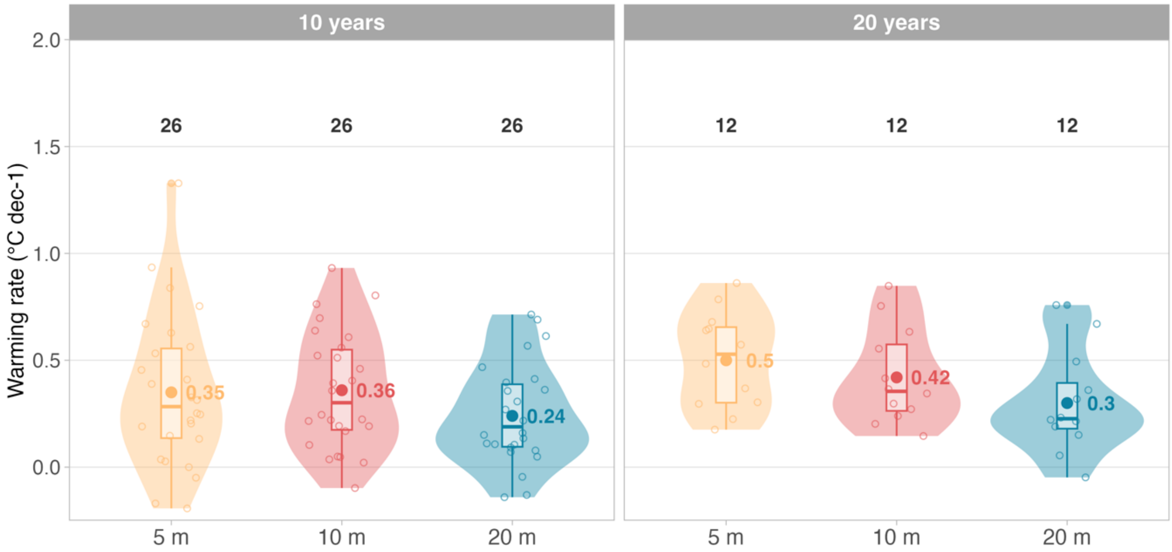

Supplemenary Fig. 5. Permafrost warming rates for the decade 2013–2022 (left) and for the 20-year period 2003–2022 (right) for three depth classes. The distribution of warming rates for each depth class (5 m, 10 m, and 20 m) is shown by violins and overlaying boxplots, individual data points are shown by circles and mean values are shown by filled points and in °C dec^{-1} next to them. The number of time series for each class is given at the top. Only time series in permafrost and with data available for all three depths are shown here.

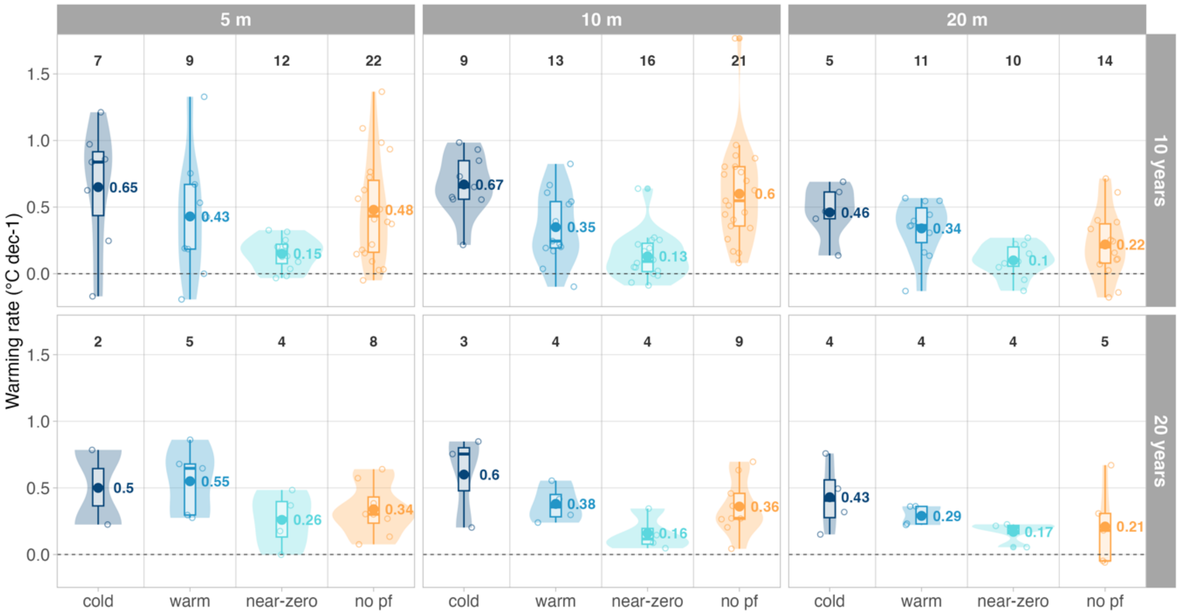

Supplemenary Fig. 6. Permafrost warming rates classified by depth and permafrost conditions. Warming rates of ground temperatures for the 10-year period 2013–2022 are shown in the upper panels and for the 20-year period 2003–2022 in the lower panel, the three depths classes are shown from left to right. In all panels, the distribution for each class is shown by violins and overlaying boxplots, individual data points are shown by circles, and mean values are shown by filled points and in °C dec^{-1} next to them. The number of time series for each class is given at the top. Zero warming is highlighted by the dashed horizontal line.

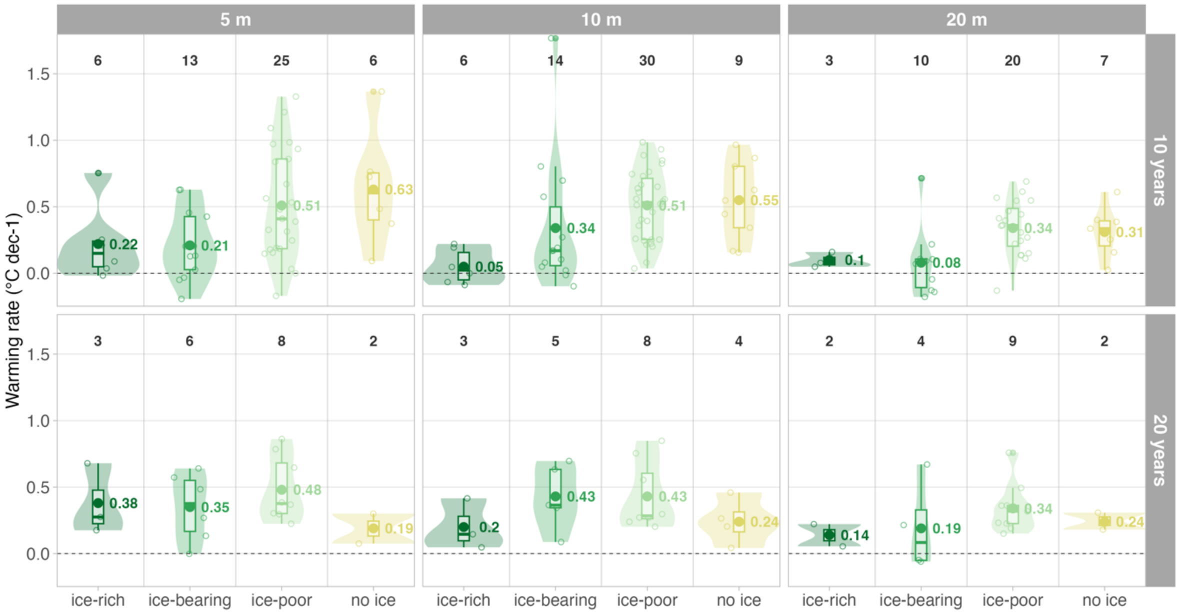

Supplemenary Fig. 7. Permafrost warming rates classified by depth and ground ice content. Warming rates of ground temperatures for the 10-year period 2013–2022 are shown in the upper panels and for the 20-year period 2003–2022 in the lower panel, the three depths classes are shown from left to right. In all panels, the distribution for each class is shown by violins and overlaying boxplots, individual data points are shown by circles, and mean values are shown by filled points and in °C dec^{-1} next to them. The number of time series for each class is given at the top. Zero warming is highlighted by the dashed horizontal line.

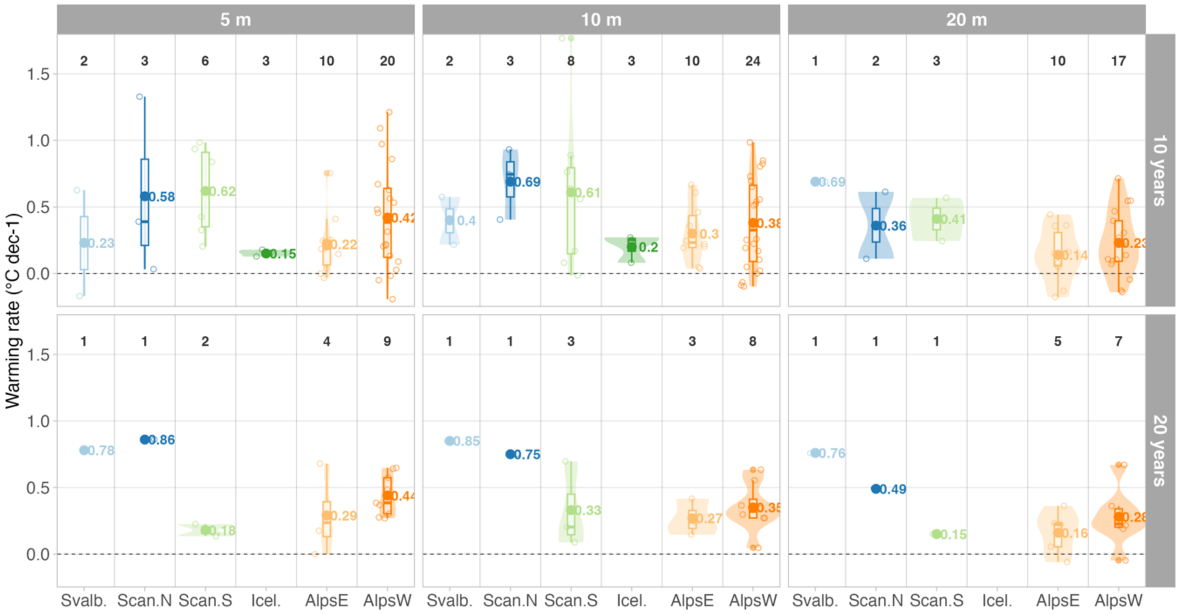

Supplemenary Fig. 8. Permafrost warming rates classified by depth and six European mountain regions (Svalbard, Scandinavia North, Scandinavia South, Iceland, Eastern Alps and Western Alps). Warming rates of ground temperatures for the 10-year period 2013–2022 are shown in the upper panels and for the 20-year period 2003–2022 in the lower panel, the three depths classes are shown from left to right. In all panels, the distribution for each class is shown by violins and overlaying boxplots, individual data points are shown by circles, and mean values are shown by filled points and in °C dec^{-1} next to them. The number of time series for each class is given at the top. Zero warming is highlighted by the dashed horizontal line.

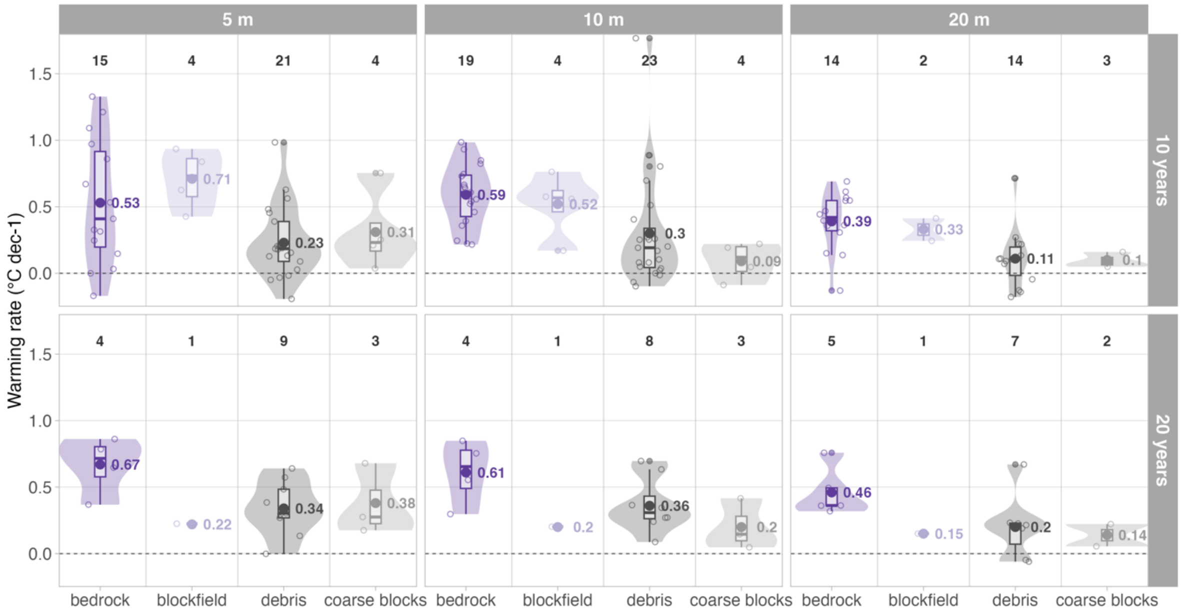

Supplemenary Fig. 9. Permafrost warming rates classified by depth and surface cover. Warming rates of ground temperatures for the 10-year period 2013–2022 are shown in the upper panels and for the 20-year period 2003–2022 in the lower panel, the three depths classes are shown from left to right. In all panels, the distribution for each class is shown by violins and overlaying boxplots, individual data points are shown by circles, and mean values are shown by filled points and in °C dec^{-1} next to them. The number of time series for each class is given at the top. Zero warming is highlighted by the dashed horizontal line.
