# Supplementary material for: Enhanced warming of European mountain permafrost in the early 21st century
Source: Nat Commun. 2024 Dec 10;15:10508. doi: 10.1038/s41467-024-54831-9 (PMC11631975; doi:10.1038/s41467-024-54831-9)
Supplement: Supplementary file 2 — Description of Additional Supplementary Files [file 41467_2024_54831_MOESM2_ESM.pdf]

Description of Additional Supplementary Files

File Name: Supplementary Data 1
Description: ***Metadata for sixty-four ground temperature time series in European mountain permafrost regions:*** *Information on the location, characteristics and responsible program or institution for the ground temperature time series included in the study.*
